# Potential Benefits, Limitations and Target Product-Profiles of Odor-Baited Mosquito Traps for Malaria Control in Africa

**DOI:** 10.1371/journal.pone.0011573

**Published:** 2010-07-14

**Authors:** Fredros O. Okumu, Nicodem J. Govella, Sarah J. Moore, Nakul Chitnis, Gerry F. Killeen

**Affiliations:** 1 Biomedical and Environmental Sciences Thematic Group, Ifakara Health Institute, Ifakara, United Republic of Tanzania; 2 Public Health Entomology Group, Department of Infectious Diseases, London School of Hygiene and Tropical Medicine, London, United Kingdom; 3 Vector Group, Liverpool School of Tropical Medicine, Liverpool, United Kingdom; 4 Department of Epidemiology and Public Health, Swiss Tropical and Public Health Institute, Basel, Switzerland; BMSI-A*STAR, Singapore

## Abstract

**Background:**

Traps baited with synthetic human odors have been proposed as suitable technologies for controlling malaria and other mosquito-borne diseases. We investigated the potential benefits of such traps for preventing malaria transmission in Africa and the essential characteristics that they should possess so as to be effective.

**Methods and Principal Findings:**

An existing mathematical model was reformulated to distinguish availability of hosts for attack by mosquitoes from availability of blood *per se*. This adaptation allowed the effects of pseudo-hosts such as odor-baited mosquito traps, which do not yield blood but which can nonetheless be attacked by the mosquitoes, to be simulated considering communities consisting of users and non-users of insecticide-treated nets (ITNs), currently the primary malaria prevention method. We determined that malaria transmission declines as trap coverage (proportion of total availability of all hosts and pseudo hosts that traps constitute) increases. If the traps are more attractive than humans and are located in areas where mosquitoes are most abundant, 20–130 traps per 1000 people would be sufficient to match the impact of 50% community-wide ITN coverage. If such traps are used to complement ITNs, malaria transmission can be reduced by 99% or more in most scenarios representative of Africa. However, to match cost-effectiveness of ITNs, the traps delivery, operation and maintenance would have to cost a maximum of US$4.25 to 27.61 per unit per year.

**Conclusions and Significance:**

Odor-baited mosquito traps might potentially be effective and affordable tools for malaria control in Africa, particularly if they are used to complement, rather than replace, existing methods. We recommend that developers should focus on super-attractive baits and cheaper traps to enhance cost-effectiveness, and that the most appropriate way to deploy such technologies is through vertical delivery mechanisms.

## Introduction

The interactions between mosquitoes and humans are central to the transmission of human malaria and other mosquito borne pathogens. Blood-seeking mosquito vectors identify humans from more than 30 meters away by detecting and following the chemical cues that the humans emit [1], [2]. In recent years, studies of the olfactory mechanisms of the *Anopheles* mosquitoes, which transmit malaria in Africa, have yielded considerable insights into the molecular and physiological processes involved [3]. In some studies, the aim has been to discern how these processes influence malaria transmission [4], [5], while in others it has been to find synthetic compounds that attract or repel mosquitoes [6]–[9]. From a public health point of view, the primary motive for investigating these issues lies in the potential to create new mosquito surveillance and abatement technologies.

While their applications in public health are still limited, odor-baited technologies are widely exploited in the agricultural sector where pest control is generally more advanced than is the case for vectors of human diseases [10]. Notable examples of success include the push-pull strategies practiced in crop pest management [11]–[13] and the control of tsetse flies, which transmit human and animal trypanosomiasis [14]–[16]. In both cases, the behavior of the pest is manipulated such that, instead of finding their intended hosts, they are lured into traps or onto insecticide-treated targets. Several types of odor-baited mosquito traps have been developed but they are used primarily for sampling, rather than controlling vector populations. Common examples include traps baited with whole humans [17]–[21], and those baited with carbon dioxide or other synthetic host cues [22]–[27]. Perhaps the most convincing examples of what may be possible by introducing lethal traps or targets is provided by the most successful existing methods of malaria control today: Insecticide-treated nets (ITNs) [28], [29] and the application of indoor-residual sprays (IRS) to houses [30], [31]. Both methods essentially turn existing blood resources (people) and associated resting site resources (human dwellings) into lethal mosquito traps.

One important factor to consider before introducing new vector control methods, such as odor-baited mosquito traps, in Africa is the ongoing scale up of long lasting insecticidal nets (LLINs) across the continent [29]. These nets have lowered malaria burden in many endemic countries [28], [32], [33] and are currently prioritized as the frontline malaria prevention method across most of Africa [34]–[36]. Moreover, past and recent trends indicate that many countries are steadily increasing coverage with ITNs [29], [37]. With these developments, it is necessary that any new tools are not evaluated in isolation, but rather on the basis of how much additional benefit they confer upon these communities where nets are already being used. The successful rollout of ITNs also poses new challenges by selectively suppressing transmission by indoor biting mosquitoes that prefer human blood [38]. New complementary vector control strategies that target the more zoophagic, exophagic vector species are required to tackle the residual transmission mediated by such modified vector populations.

While some relatively expensive designs have been proposed as being suitable for trapping mosquitoes in numbers sufficient to achieve population control [25], [27], [39], [40], no rigorous large scale and independent evaluations of these technologies have been reported. More importantly, even though there is a constantly growing interest in odor-baited technologies, essential characteristics which they should posses so as to effectively control or disrupt malaria transmission have not been determined. Also unknown are the optimal approaches that could be used to deliver them as public health commodities. Nevertheless, recent field trials of novel synthetic odor blends have shown that they can exceed the attractiveness of humans by up to four fold [41] and affordable, practical outdoor trap designs are becoming available [40], [42], so the possibility of controlling malaria vector populations and malaria transmission is becoming increasingly realistic.

Here, the potential for using odor-baited mosquito traps to control malaria in a number of common epidemiological scenarios in Africa is mathematically investigated. Firstly, we examined whether traps, when used alone or as a complementary intervention alongside insecticidal nets, can fully reduce malaria transmission in highly endemic areas. Secondly, the target product-profiles that developers of this technology should consider so as to ensure effectiveness under real-life operational conditions were elucidated. These were accomplished by modifying an existing mathematical model of malaria transmission [43], which has previously been useful for informing global ITN coverage policy [36], but for which substantive revision was prompted by this particular example of odor-baited mosquito traps. The traps were treated as pseudo-hosts, which unlike humans or cattle, cannot provide blood to host-seeking mosquitoes, but which mosquitoes can attack nonetheless. This conceptual reformulation enabled explanation of the potential value and target product profiles of mosquito traps as a means to complement ITNs.

## Methods

### Description of the model

This is an adaptation of a deterministic model representing the most important host-seeking, survival and malaria transmission processes that individual mosquitoes undertake before they can transmit malaria [43]. All parameter symbols and their meanings are outlined in tables 1 and 2. Versions of the original model have been used to explore effects of bednets, cattle, repellents and insecticides on malaria transmission [44], to outline global coverage targets [36] and likely efficacy of ITNs [45], and also to examine interactions within push-pull strategies such as combining net-use with zooprophylaxis using cattle [46].

**Table 1:** Symbols and their meanings.

Symbol	Definition	References
*a*	Availability of individual hosts: rate at which a single mosquito encounters and then attacks a given single host or pseudo-host.	This paper.
*A*	Total availability of hosts and pseudo hosts: rate at which a single mosquito encounters and attacks all hosts and pseudo hosts.	This paper.
*β*	The mean number of infectious bites per emerging mosquito during its lifetime.	[43], [44], [73].
*c*	Cattle.	[43], [44].
*C_A_*	Proportion of the total available host resources accounted for by the odor-baited traps, equivalent to trap coverage.	This paper.
*C_h_*	Proportion of people using ITNs, equivalent to ITN coverage as surveyed by its most relevant indicator [117].	[43], [44].
*Δ*	Probability that a mosquito which encounters a host will be diverted from that host.	[43], [44].
*ε*	Host-encounter rate: rate at which a single host-seeking mosquito encounters a given single hosts.	[43], [44], [54].
*E*	Emergence rate of mosquito vectors per year.	[43], [44], [73].
*EIR*	Entomological inoculation rate (mean number of infectious bites that an average individual human receives per year).	[43], [44], [54], [73], [77].
*φ*	Probability that a mosquito which attacks a host will successfully feed upon that host.	[43], [44], [54].
*f*	Feeding cycle length: measured as the number of days it takes a single mosquito to get from one blood feed to the next.	[43], [73].
*g*	Gestation interval: number of days a mosquito takes to digest a blood meal and return to searching for oviposition site.	[43], [44].
*h*	Humans.	[43], [44].
*h,p*	Protected humans using ITNs.	[43], [44].
*h,u*	Unprotected humans not using ITNs.	[43], [44].
*κ*	Human infectiousness to mosquitoes: probability of a vector becoming infected per human bite.	[43], [49], [73].
*λ*	Relative availability of hosts other than humans: calculated as a ratio of availability of those hosts to availability of humans not using ITNs.	[41], [43], [54].
*L*	Potential of any individual vector to transmit malaria from infectious humans over its lifetime.	[73].
*μ*	Probability that a mosquito which attacks a host will die during the attack.	[43], [44].
*η_o_*	Oviposition site-seeking interval: number of days that a mosquito takes to find an oviposition site once it starts searching for it.	[43], [44].
*η_v_*	Host-seeking interval: number of days a mosquito takes to find and attack a host.	[43], [44], [54].
*N*	Number of hosts.	[43], [44].
*θ_Δ_*	Excess proportion of mosquitoes which are diverted while attempting to attack a human while that person is using an ITN.	This paper.

**Table 2:** Symbols and their meanings-continued from table 1.

Symbol	Definition	References
*θ_μ_*	Excess proportion of mosquitoes which die while attempting to attack a human while that person is using an ITN.	This paper.
*Ω*	Intervention package scenarios consisting of a specific coverage with ITNs and a specific number of odor-baited mosquito traps per 1000 people.	This paper.
*π_i_*	The proportion of normal exposure to mosquito bites upon humans lacking ITNs, which occurs indoors at times when nets would normally be in use.	[43], [45], [68].
*P*	Probability that a resting mosquito survives any one day.	[43], [44].
*P_f_*	Probability that a mosquito survives a single complete feeding cycle.	[43], [44].
*P_ov_*	Probability that a mosquito survives any full day of the oviposition site-seeking interval or host-seeking interval.	[43], [44].
*Q_h_*	Human blood index: the proportion of all blood meals from all hosts and pseudo hosts, which are obtained from humans.	[43], [44], [54], [73].
*s*	Host species or host type	[43], [44].
*t*	Odor-baited mosquito traps.	This paper.
*γ*	Probability that a mosquito attacks an encountered host.	
*ψ*	Relative exposure of different hosts other than unprotected humans to mosquito bites: calculated as a ratio of exposure of those hosts to exposure of humans not using nets.	This paper.
*ψ_h,p,Ω_*	Combined personal and communal protection provided by the integrated intervention package *Ω* to people who use ITNs.	This paper.
*ψ_h,Traps_*	Additional protection offered by odor-baited traps to communities using ITNs.	This paper.
*ψ_h,u,Ω_*	Communal protection provided by the integrated intervention package Ω to people who do not use ITNs.	This paper.
*ψ_Ω_*	Mean relative exposure of an average member of a community where the intervention package *Ω* is implemented.	This paper.
*z*	Availability of blood from an individual host: rate at which a single mosquito encounters, attacks and successfully feeds upon a given single host	This paper.
*Z*	Total availability of blood from hosts and pseudo hosts: rate at which a single mosquito encounters, attacks and successfully feeds upon all hosts.	This paper.

**Figure 1:**
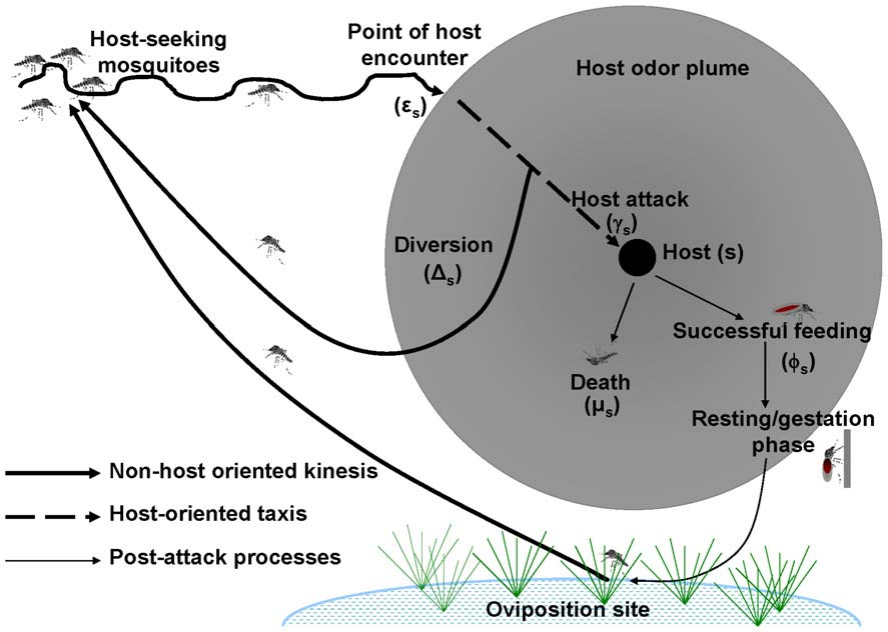
A simplified conceptual structure of the adapted model. This figure shows behavioral and mortality processes that occur in a mosquito feeding cycle. The host-seeking process includes *non-host oriented kinesis* and *host-oriented taxis*. The gray circular area represents extent of detectable odor plume around a host of species or type (s). In blood acquisition processes, mosquitoes are said to encounter hosts when they first detect odor cues associated with that host (ε_s_). Then they can either attack the encountered host (γ_s_) or be diverted back to non-host-oriented kinesis (Δ_s_). Mosquitoes which go on to attack the host can either successfully feed (φ_s_) or die (μ_s_). Mosquitoes which successfully feed will go on to rest, digest the blood meals and then oviposit their eggs before eventually returning to host seeking state. This diagram is not drawn to scale and the host odor plume may not always be circular.

Blood feeding is the most important epidemiological event in the interactions between humans and malaria vector mosquitoes [47], [48]. In this model, the blood acquisition process is considered as having three phases: 1) the mosquito being in a host-seeking state, 2) the mosquito attacking the host (or diverting away) and 3) the mosquito feeding upon the host (Fig. 1). As in previous works by other authors, this feeding process is considered to be cyclical rather than continuous, so as to more accurately represent natural events [49], [50]–[52]. The model examines diversion and mortality processes that occur during the three phases and how changes induced by interventions upon these processes can contribute to individual and community-level protection against malaria.

Effects of odor-baited traps were simulated in conceptual environments of two alternative dominant vector species (*Anopheles gambiae sensu stricto* Giles or *An. arabiensis* Patton) [53] in the presence of cattle, the main alternative blood source for these vectors [54], and presence or absence of ITNs. In each test scenario, the technology was evaluated in terms of combined, individual and community-level protection against malaria transmission when traps are implemented alone or in combination with ITNs.

Similar to most malaria transmission models, an enclosed ecosystem of parasites, vectors and hosts, is assumed [55], [56]. In order to further reduce computational complexity, the human hosts are considered to be homogenously mixed, meaning that vulnerability of individuals to malaria infection [5], [57] or attractiveness of individuals to mosquitoes [2], [58], [59] can be reasonably estimated using population mean values for these parameters. These assumptions allowed for exploration of what might be possible if the traps are concentrated in geographical areas where mosquito densities are most abundant. Such locations are known to exist in real field settings [60]–[62] and can be targeted to achieve greatly enhanced control of pathogen transmission [63].

In the original model, the term ‘hosts’ referred to any vertebrate blood-sources upon which vectors can feed. This definition is hereby expanded to include all entities that a vector can attack with the intention of taking a blood meal, regardless of whether that entity actually has blood or not. This redefinition allows for inclusion of odor-baited traps as additional hosts (more precisely, pseudo-hosts) even though mosquitoes cannot possibly obtain blood from them. Another modification was a more explicit sub-division of the host-seeking process. Unlike the original model, the host-seeking process is considered here as consisting of two successive stages leading to the mosquito attacking the host namely: 1) non-host oriented *kinesis*, referring to arbitrary movements of the mosquito before it detects host cues, a process which ends with a host encounter event, and 2) host-oriented *taxis*, referring to directional movements of the mosquito once it encounters and detects the host cues in the environment and starts moving towards the source of those cues, a process which if initiated, either ends with a host attack event, or is aborted resulting in diversion back to *kinesis* (Fig. 1).

The duration of non-host-oriented kinesis, which is equivalent to the reciprocal of the rate at which an individual host is encountered by an individual vector, depends on: 1) physical distance between hosts and mosquitoes and 2) the distance over which attractive host odor plumes can extend. This means mosquitoes are more likely to encounter hosts which are near to the point at which they began host-seeking than those hosts which are far away. In nature, such spatial relations, including modifiers such as topography and wind direction are known to be important determinants of rates at which individual hosts are encountered [60]–[65]. This definition of the kinesis process also means that mosquitoes will more readily encounter hosts whose odor plumes extend over a wide radius than hosts which have short-radius plumes. For the purposes of this model, wider odor plumes are regarded as being equivalent to more mosquitoes potentially falling within the range of host encounter. Therefore hosts generating such kairomonal plumes are considerably more readily available than hosts generating less dispersed, short radius plumes. Interestingly, recent field trials of odor-baited traps demonstrate that the host-specific cues which malaria vector mosquitoes use to identify their preferred human hosts act mainly as long range attractants, presumably triggering the encounter process itself and allowing mosquitoes to make the choice between attack and diversion as early and as efficiently as possible [41].

Host-oriented taxis begins immediately after host encounter once the mosquito as chosen to proceed with host attack. There is a possibility that a mosquito encountering a non-preferred host type will ignore the opportunity to approach the host or may discontinue taxis, thus diverting back to non-host-oriented kinesis to seek other hosts. Once the mosquito commits to attack a host, it is assumed to complete a full taxis phase which ends with the host attack event.

The original definition of host availability [43] was also altered to specifically and separately describe the availability of hosts for attack rather than availability of host blood *per se*. The availability (*a*) of any host of any species or type (*s*) for mosquitoes to attack is the product of the rate at which individual vectors encounter that host (ε_s_) and the probability that, after this encounter, they will attack the host (γ_s_):
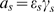
(1)Previously, host availability had been described as the product of host encounter rate and feeding probability [43], [44], [46], [54]. Replacing the term, *feeding* with the term, *attack*, allows us to model the behavior of mosquitoes which attack the odor-baited traps and for which the feeding probabilities are therefore nil. A closer examination of what was previously defined as host availability [43] reveals that actually, it represents the availability of host blood at a particular source rather than the availability of the hosts themselves. That is to say, the availability of host blood (*z*) from a host of any species or type (*s*) is the product of the rate at which individual vectors encounter this host (*ε_s_*) and the probability that, after this encounter, they will successfully feed upon that particular host (*φ_s_*):
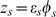
(2)


Similar to the original model, we label certain parameters with subscript *s* to represent different host species or host types including humans, cattle or odor-baited traps. Also, where necessary, the subscript *s* is specified as one of three different subscripts, *t*, *c*, *h* to represent traps, cattle and humans respectively. Moreover, humans not using nets (unprotected humans) and humans using nets (protected humans) are in some cases specifically represented by subscripts *h,u* and *h,p* respectively. Another subscript, *j*, which was used in previous versions of the original model [43], [44] to represent individuals within different host types or species, has been omitted in this reformulation, as no specific individual hosts are considered and instead, all parameters in this paper represent mean values for respective host populations.

When the mosquito encounters the host, it can either attack the host (successfully completing the host-seeking process, but not necessarily the blood acquisition process) or it can be diverted from the host (aborting the host-seeking process). The attack (*γ_s_*) and diversion (Δ_s_) probabilities therefore sum to unity.
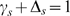
(3)


After host encounter, all diverted mosquitoes are assumed to re-enter non-host-oriented kinesis afresh. The diversion may include behavioral responses of mosquitoes to non-preferred or protected hosts which prompt them to abort taxis. For preferred hosts, diversion may be induced by physical barriers like house screens and untreated nets or chemicals used to treat nets or houses, and which repel or irritate mosquitoes [66], [67].

However, not all vectors that attack the host will successfully feed. To account for mosquitoes that die during this attack process, a term for the mean attack-related mortality (*μ_s_*) is introduced. It is assumed that only two possibilities exist at this stage: either the vector feeds successfully and consequently survives or it dies in the attempt before obtaining a blood meal. All mortality risks associated with host attack are expressed as a single mean probability and assumed to occur prior to feeding. The probability of successful feeding per host encounter (*φ_s_*) is therefore calculated as follows:
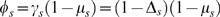
(4)Assuming similar levels of baseline host defensiveness, the probabilities of diversion (Δ) and attack related mortality (*μ*) are considered to be same for cattle (*c*) and humans who are not using ITNs, i.e. unprotected humans (*h,u*). Equation 4 can therefore be specified as follows:
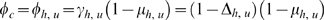
(5)


Personal and house-hold protection measures such as bednets, repellents or domestic insecticides function by diverting host-seeking vectors or killing the vectors. The terms, Δ and *μ* are therefore modified for ITN users i.e. protected humans (*h,p*), to become Δ*_h,p_* and *μ_h,p_* respectively. Consistent with Killeen & Smith (2007) [44], the new terms are obtained by adding the ITN-induced changes to the baseline diversion and baseline mortality values:
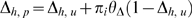
(6)

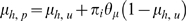
(7)Where, *θ_Δ_* and *θ_μ_* represent the additional effects of ITNs on the diversion and mortality probabilities respectively. These coefficients were previously annotated as Δ*_p_* and μ*_p_* in the original model [43], [44] but have now been changed to distinguish them more clearly from the Δ*_h,p_* and Δ*_h,u_*, which refer to diversions from protected and unprotected humans respectively. The term *π_i_* in the two equations refers to the proportion of normal exposure to mosquito bites upon humans lacking ITNs that occurs during the times when nets would normally be in use [45], [68]. It is used here to modify the terms *θ_Δ_* and *θ_μ_*, in order to obtain the true effects of ITNs upon a typical user. Without the term, *π_i_*, the equations would represent merely an ideal situation where ITNs are consistently and correctly used over the full course of the time when malaria vectors bite. However, such an ideal scenario seldom happens and possessing a net does not always translate to consistent and perfect use of it. Moreover, even the most nocturnal vectors can feed to some extent in the early evening hours before people go under their nets or in early mornings when many people are awake and are no longer protected [45], [67], [68]. Thus in practice, not all human exposure to mosquito bites occurs during the times when nets are actually in use [45], [67]–[69]. Note that this approach deals more simply and parsimoniously with such behavioral avoidance of interventions, than previous approaches by incorporating these effects at the single point of the model where they actually act in biological reality, rendering the more elaborate and indirect formulations such as equation 8 in Killeen *et al.*, 2007 [43] and equation 1 in Govella *et al.*, 2010 [45], redundant.

Equations 6 and 7 are used to specify equation 4 in order to explicitly express the probability of successful feeding upon an ITN user (*φ_h,p_*):
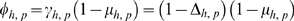
(8)


### Modeling the effects of individual odor-baited traps

Odor-baited traps are assumed to affect the foraging behavior of host-seeking mosquitoes by triggering the transition from kinesis to taxis, in exactly the same way as vertebrate hosts. Their efficacy as tools to control malaria transmission is derived primarily from two complementary characteristics: 1) their high attractiveness to malaria mosquitoes compared to attractiveness of humans [41] and 2) their ability to trap and kill mosquitoes which attack them thus removing these mosquitoes from the biting population. Any given trap type can therefore be described in terms of its mean availability for attack by host-seeking mosquitoes (*a_t_*), defined as the rate at which it is encountered (*ε_t_*), and the probability that it is attacked by the mosquitoes (*γ_t_*) following encounter. As successful blood feeding upon a trap is not a possible outcome, the mortality probability for mosquitoes that attack a trap (*μ_t_*) and the corresponding probability of successful blood feeding (*φ_t_*), are fixed at one and zero respectively (*μ_t_* = 1, *φ*_t_ = 0).

These assumptions about individual-level processes enable adaptation of subsequent equations from the original formulation [43], so as to estimate population-level effects of odor-baited traps used alone or in combination with ITNs, and also to elucidate desirable characteristics of such devices.

### Estimating population level effects of odor-baited traps when used alone or in combination with ITNs

The availabilities of cattle (*a_c_*) and traps (*a_t_*) for attack by host-seeking mosquitoes were calculated based on field estimates of their relative availabilities (*λ_c_* for cattle [54] and *λ_t_* for odor-baited traps [41]) when compared to the availability of humans for similar attacks as described in equation 1:
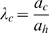
(9)

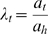
(10)


For any given number of odor-baited traps (*N_t_*), cattle (*N_c_*), people not using ITNs (*N_h,u_*) and people using ITNs (*N_h,p_*), the total host availability (*A*) was calculated as the sum of the products of mean availabilities of each host species or type (*a_s_*) and the number of hosts of that particular species or type (*N_s_*). However, unlike in the original formulation [43], the term host availability hereby includes events only up to host attack, thus excluding all probabilities of blood feeding or death after the attack. The mean host-seeking interval (*η_v_*) was then calculated as the reciprocal of total host availability (*A*) and consistent with previous formulations [54]:
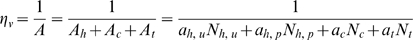
(11)


The relative exposure of any host to mosquito bites (which is calculated as a function of successful feeding and therefore the availability of blood rather than hosts *per se*) is therefore no longer equivalent to its relative availability when calculated as a function of host attack probability. This means that any two hosts can be equally available for attack but may be differentially exposed if interventions which cause different levels of reduction of successful feeding despite equal levels of diversion are specified. The relative exposure *(ψ)* of different hosts must therefore be calculated separately from relative availability of attackable hosts and must be based on the availability of the blood resource that each host type or species (*s*) represents to mosquitoes (*z_s_*). For example, relative exposure of humans protected with ITNs, when compared to that of humans not protected with ITNs is calculated as follows:
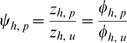
(12)where *z_h,p_* refers to the mean availability of blood from a protected human.

For a vector to complete one feeding cycle, it must survive all the host-seeking phases shown in figure 1 including gestation to convert blood to eggs and then an equivalent set of resource acquisition processes required to enable oviposition. While gestation is primarily spent resting in relatively safe places, which are often inside houses, foraging for resources is an intrinsically dangerous process for mosquitoes. Even without any human intervention, survival is reduced by numerous biotic and abiotic factors in the environment such as predators, host defensive behavior and dehydrating conditions of heat and low humidity [70], [71].

As in our original model [43] and in some previous models by other authors [50], [72], it was assumed that survival during host-seeking and oviposition site-seeking phases is lower than survival while the mosquito is resting inside houses. Survival across all phases of the gonotrophic cycle was estimated as the distinct daily survival probability during each phase to the power of the respective time intervals, namely the host-seeking interval (*η_v_*), gestation interval (*g*) and oviposition site-seeking interval (*η_o_*). Though the current definition for host-seeking refers to processes up to and including attack, but not blood acquisition itself, the duration between the time when the mosquito attacks the host and the time when it bites and acquires blood from it, is considered here to be a negligible interval in the context of a gonotrophic cycle which lasts for two or more days. The daily survival probability of a resting mosquito is defined as P and the survival probabilities during host-seeking and oviposition site-seeking are assumed to be equal and are both defined using the term (*P_ov_*). The survival rate per feeding cycle (*P_f_*) was therefore estimated as the combined probability that a vector survives gestation (*P^g^*), oviposition site-seeking (*P_ov_^ηo^*), host-seeking (*P_ov_^ηv^*) and the eventual attack of a host (*P_γ_*):
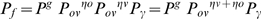
(13)


To calculate the probability of mosquitoes surviving their eventual attack upon any host (*P_γ_*), we assumed that the proportion of all attacks that end in death is the mean of the mortality probabilities for attacking the various hosts (non-ITN users, ITN users, cattle or odor-baited traps), weighted according to the proportion of total availability that each host class represents [45]:
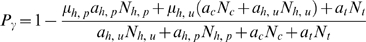
(14)


This term differs slightly from equation 13 of the original formulation [43], in that it now reflects ITN effects that have been modified by the proportion of normal unprotected human exposure that occurs during times when this intervention would typically be in use (*π_i_*) [45], [68], but does so more directly than the more complex formula of Govella *et al.*, 2010 [45] because this effect has already been captured by equations 6 and 7. The term for mortality upon attacking an odor-baited trap (*μ_t_*) could be included explicitly in the numerator so that the equation is clearer, but because it has already been defined as being equal to one, the trap terms in both the numerator and denominator are expressed simply as *a_t_N_t_*. Here again, this revised formulation is more specific and predicts survival of attack based only on rates of attack rather than the probabilities of successful feeding.

The human blood index (proportion of all blood-meals that originate from humans; *Q_h_*), was calculated based on the proportion of the total availability of blood from all host types (*Z*), which humans represent (*Z_h_*). Note that for any host species or type, *Z_s_* = *z_s_N_s_*. Specifically, *Q_h_* was therefore calculated as the proportion of surviving mosquitoes obtaining a blood meal that do so from humans, based upon the overall total rates of encounter of each host type and the probabilities of successfully obtaining a blood meal from each:
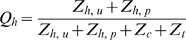
(15)

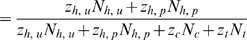
(16)

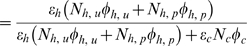
(17)It should be noted that equation 17 also does not contain terms for odor-baited traps (*N_t_*, *ε_t_* and *φ_t_*) in the denominator. This is because it is impossible for mosquitoes to obtain blood meals from the traps so even if the term *φ_t_* were included, it would be valued zero thus rendering the equation mathematically equivalent to the above.

### Estimating protection against exposure to malaria

As described in the very first formulation of the population-level component of this hierarchical model [73] and its subsequent improvements [43], [44], the survival rate per feeding cycle (*P_f_*) and the proportion of blood meals taken from humans (*Q_h_*) were used to calculate the potential of any individual vector to transmit malaria from infectious humans over its lifetime (*L*). The term L together with human infectiousness to mosquitoes (*κ*) were then used to calculate the mean number of infectious bites per emerging mosquito during its lifetime (*β*). To obtain the sum of all infectious bites that occur in the whole human population, the mean number of infectious bites per emerging mosquito (*β*) was multiplied by the emergence rate of mosquito vectors (*E*). If this product (*βE*) is divided by the human population size (*N_h_*), we obtain the mean number of infectious bites that an average individual human receives, also referred to as the mean entomological inoculation rate (EIR) experienced by individuals in the community [73], [74]:
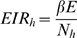
(18)


In a human population composed of two distinct subgroups (ITN users and non-users), it is important to calculate separately the EIR experienced by each subgroup so that we can compare them. For either subgroup, this is a product of the total number of infectious bites upon humans that occur in the population as a whole (*βE*) and the fraction of biting exposure experienced by that particular subgroup of the population. Here also, the original forms of these equations [43] are replaced with explicit forms to express the availability of blood rather than the availability of attackable hosts, and consequently capture exposure to bites rather than exposure to attacks:
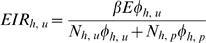
(19)

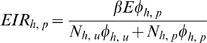
(20)


For purposes of estimating the likely impacts of interventions, it is imperative to know how much the exposure to bites from malaria-infected mosquitoes can change when an individual becomes protected by a preventative measure such as an ITN. Dividing equation 20 by equation 19 and substituting with equation 12 provides a solution which is consistent with the commonly accepted definition of personal protection against exposure to infectious bites [68], [75]:
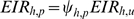
(21)


For integrated programs, involving the use of ITNs and odor-baited traps, there are several possible intervention package scenarios (*Ω*). Each package is explicitly defined by the ITN coverage (*C_h_*), ITN properties (*θ_Δ_* and *θ_μ_*), number of odor-baited traps (*N_t_*) and the mean availability (*a_t_*) of those traps. For ease of comparison and interpretation, the impact of any intervention package, *Ω*, is expressed in terms of relative exposure to transmission intensity (*ψ_Ω_* = *EIR_Ω_/EIR_0_*), where *EIR_Ω_* is the mean exposure of humans in the presence of the intervention package and *EIR_0_* is the mean exposure of members of the same community when no intervention is present. We use the notation *EIR_0_* = *EIR_h,u,0_* to denote the EIR of all humans when no intervention is present, *EIR_h,u,Ω_* to denote the EIR of humans without ITNs in a population with the intervention *Ω* and *EIR_h,p,Ω_* to denote the EIR of humans with ITNs in a population with the intervention *Ω*. The mean EIR in the presence of the intervention package is therefore:
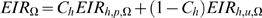
(22)where *C_h_* is the proportional coverage of the human population with ITNs.

The total benefits of any intervention package, *Ω* can then be apportioned to personal or communal protection benefits and expressed in terms of EIR relative to the baseline scenario with no interventions as follows:
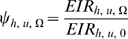
(23)for communal protection provided by the integrated intervention package to people who do not use ITNs, and
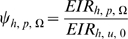
(24)for combined personal and communal protection provided by the integrated intervention package to people who use ITNs.

Whereas people who do not use ITNs will benefit from only the communal protection provided by the integrated intervention package, those who use ITNs will benefit from both the personal protection provided by their own ITNs and the communal protection provided by the integrated intervention package. The contributions of personal and community-level protection to the benefits of ITNs have been discussed in detail elsewhere [43] and are therefore not the focus of this paper. Here, we express the influence of ITNs simply as the mean relative exposure of an average member of the community. This is calculated as the mean of the relative *EIR* of protected and unprotect hosts, weighted according to the proportions of the human population that they represent:
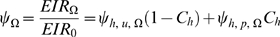
(25)


When odor-baited traps are added to the intervention package alongside ITNs, we expect that the exposure of both net users and non-users to infectious mosquitoes is correspondingly reduced. Because ITNs are already widely used in Africa [29], the traps should be considered only as complementary interventions rather than as replacement for the ITNs. Their effects on transmission should therefore be evaluated in terms of the further transmission reductions they offer, relative to that which is provided by ITNs alone. To determine how much benefit the odor-baited traps would actually contribute towards the overall reductions generated by the combined intervention, the residual exposure experienced when the combined package is implemented is expressed relative to the residual exposure experienced when only nets at any given coverage (*C_h_*) are used:
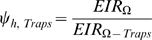
(26)reflecting additional protection offered by odor-baited traps to communities using ITNs.

Because odor-baited traps are considered as a distinct host type (more specifically pseudo-hosts), we used this model to explore the hypothesis that their effects on malaria transmission will depend on how much they contribute to the total availability of all hosts for attack by malaria mosquitoes, which is equivalent to the proportion of the total available host resources covered or accounted for by the odor-baited traps (*C_A_*):
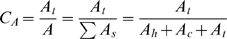
(27)


It is expected that as *C_A_* increases, so will the impact of the traps on malaria transmission. With reference to these reformulated equations there are two possible ways to increase total trap availability (*A_t_*) and therefore increase *C_A_*. These include increasing the relative availability of individual traps (*λ_t_*) or increasing the number of traps deployed (*N_t_*). Similarly, with reference to the current definition of mosquito host-seeking processes, the relative availability of individual traps (*λ_t_*) can be increased by ensuring high encounter rates and high attack probabilities relative to that of the preferred vertebrate hosts such as cattle and humans. Practical ways to effect such enhancements are outlined explicitly in the section entitled *parameters describing odor-baited traps*.

### Baseline ecological parameterization of the model

In table 3, the ecological parameters and associated values used as well as the source references are outlined. As in the original model [43], a village with 1000 persons and 1000 head of cattle is considered. Parameter value for infectiousness of humans to mosquitoes (*κ*) was also set the same as in the original model (0.030). It was assumed that infectiousness of humans to mosquitoes is constant across the population, regardless of the impacts of vector control measures. Therefore any additional benefit that may be accrued by reducing this parameter once EIR drops below the threshold of 10 infectious bites per person per year [76] is ignored. To achieve baseline transmission intensities representative of places in Africa where malaria transmission is constantly intense [77], [78], we increased the mosquito emergence rate from the original value of 9 million [43] to 20 million, which resulted in baseline EIR values greater than 200 in the test scenarios, thus a typically challenging holoendemic scenario was represented.

**Table 3:** Values and references for ecological parameters in the simulations.^a^

Definition	Symbol	Value	References
Total number of cattle	*N_c_*	1000	[43].
Total number of humans	*N_h_*	1000	[43].
Diversion probability from an unprotected vertebrate host (cattle or human)	*Δ_h,u_*	0.1	[43].
Mortality probability upon attacking an unprotected host	*μ_h,u_*	0.1	[43].
Mean availability of individual unprotected humans^b^	*a_h,u_*	1.2×10^−3^	[43], [54], [79].
Mean availability of individual cattle^c^	*a_c_*		
*An. arabiensis*		1.9×10^−3^	[43], [46], [54].
*An. gambiae s.s.*		2.5×10^−5^	[43], [46], [54].
Total availability of aquatic habitats	*A_a_*	3	[43].
Duration of gestation	*g*	2	
Proportion of mosquitoes surviving per day while feeding while resting	*P*	0.9	[43].
Proportion of mosquitoes surviving per day while foraging for hosts or oviposition sites	*P_ov_*	0.8	[43].
Duration of the parasite sporogonic development period	*n*	11	[43].
Human infectiousness to mosquitoes	*κ*	0.03	[43].
Total number of adult mosquitoes emerging per year	*E*	2.0×10^7^	This paper.

a This table contains only those ecological parameters considered to be necessary for the primary understanding and parameterization of the model. A full listing of all ecological parameters is available in tables 1 and 2 and in file S1, within the spreadsheet containing the model. All entries refer to mean parameter values in this deterministic model.

b The value of the parameter is equivalent to attacks per day per host-seeking vector per unprotected human.

c The value of the parameter is equivalent to attacks per day per host-seeking vector per individual head of cattle and was different for the two vector species *Anopheles arabiensis* and *Anopheles gambiae sensu stricto*. With the exception of this parameter, all the other values are assumed to be identical for both species.

The daily survival probability of a resting mosquito was set to 0.9 while the daily survival probability of mosquitoes while foraging for blood or oviposition sites (*P_ov_*) was set to 0.80, also consistent with published applications of the original model formulations [43], [44]. The baseline host defences of people who do not use ITNs, and of cattle, were assumed to be the same. Therefore, the probabilities for *An. arabiensis* and *An. gambiae s.s.* being diverted (Δ) or killed (*μ*) during attack on either non-ITN users or cattle was set as 0.1. This means 90% of all mosquitoes of these species would attack the hosts upon encountering them and thereafter 90% of those that attack the hosts will successfully take blood meals from them.

The mean availability of non ITN-users had been estimated for *An. arabiensis* on the basis of field estimates in a southern Tanzanian village at a time when less than 1% of the population used nets [79]. The study considered dissection based observations of the dilation status of ovariolar stalks in host-seeking female mosquitoes caught with human-baited light traps [79]. The number of successful feeds per day per host-seeking vector per human was therefore originally calculated as the inverse of the inferred host-seeking interval of 0.7 days divided by the human population size in the study area, which was 1212 at that time [80].

Reconsidering this estimate in the light of this revised definition of host availability for attack, this approach to parameterization now seems even more appropriate as the dissected unfed mosquitoes were sampled during the attack phase, before feeding and obviously before death. In fact, the availability value used in the original model should actually have been defined as successful attacks (rather than successful feeds) per day per host-seeking vector. For the purposes of this new model formulation, the parameter value therefore remains unchanged and was applied also to *An. gambiae*. The mean availabilities of humans to *An. arabiensis* and *An. gambiae* were then used to calculate the mean availability of cattle to attack by the same vector species. Based on equation 9, this was accomplished by calculating the product of these mean availabilities (*a_h_*) and estimates of the relative availability of cattle (*λ_c_*), which had earlier been derived from field studies of mosquito host preferences [46], [54]. Finally, the total availability of aquatic habitats (*A_a_*) was set to 3, also unchanged from the previous application [43].

### Parameters describing Insecticide Treated Nets

The intervention parameters and associated values used, as well as the source references, are outlined in table 4. We considered baseline scenarios to be communities lacking traps but where ITNs were either completely absent or being used by half of all age groups within the community. As in the original model, the effects of ITNs were quantified in terms of their ability to repel malaria vectors from humans and/or to kill the vectors whenever they attacked the net users. Though the World Health Organization, has to date approved seven different Long Lasting Insecticide Nets (LLINs), including interim approvals [81], we simulated scenarios with one long-lasting insecticidal net type, namely Olyset ® nets, whose properties are representative of the most commonly used LLINs in Africa. These LLINs are knitted from polyethylene fibres that have been impregnated with a first-generation synthetic pyrethroid, namely permethrin [82]–[84]. Apart from being toxic to mosquitoes, permethrin is also an excito-repellent, meaning that the nets also divert considerable proportions of these mosquitoes even before they can attack net users [82]–[87]. The parameter values used in the simulation were chosen such that they approximate the properties of Olyset ® nets under normal conditions of community use.

**Table 4:** Values and references for intervention parameters in the simulations.^a^

Definition	Symbol	Value	References
Proportion of people using ITNs.	*C_h_*	0.001^b^ or 0.5	This paper
Proportion of exposure that occurs indoors during the time when ITNs are actually in use.	*π_i_*	0.9	[43], [45], [68]
Number of odor-baited mosquito traps.	*N_t_*	varying	This paper
Additional diversions per ITN user encountered.	*θ_Δ_*	0.5	This paper
Probability of mosquitoes being diverted from an odor-baited trap.	*Δ_t_*	0.1	This paper
Probability of mosquitoes dying upon attacking an odor-baited trap.	*μ_t_*	1	This paper
Additional mortality of mosquitoes per ITN user attacked.	*θ_μ_*	0.7	This paper
Probability of mosquitoes successfully feeding upon an odor-baited trap.	*φ_t_*	0	This paper
Relative availability of odor-baited mosquito trap to host seeking mosquitoes if the traps are placed homogenously among humans.	*λ_t,unbiased_*	4	[41]
Relative increase in availability of odor-baited mosquito traps achieved by spatially biasing position of the traps on the basis of 80–20 statistical distribution [63].	*λ_t,biased_*	4	This paper

a This table contains only those intervention parameters considered to be necessary for primary understanding and parameterization of the model. A full listing of all intervention parameters is available in tables 1 and 2 and in File S1, within the spreadsheet containing the model. All values represent mean parameter values in this deterministic model.

b It is assumed that only one person among the 1000 people is using the ITNs.

Repellency of nets, which is measured as a reduction in the number of mosquitoes that enter human-occupied huts [88] when the nets are used by the occupants, is reflected in the excess diversion of mosquitoes from an ITN user (*θ_Δ_*). Correspondingly, the excess mortality upon attacking the ITN user (*θ_μ_*) is estimated as the excess proportion of mosquitoes entering those experimental huts that die attempting to feed on the hut occupants, relative to control huts. The parameter values of the selected representative net type were set to reflect the following: 1) diversion of 50% of all mosquitoes that encounter the net users (*θ_Δ_* = 0.5), and 2) excess mortality of 70% of those mosquitoes attacking the net users (*θ_μ_* = 0.7). These estimates were computed from reports of experimental hut studies previously conducted in the field [83]–[85], [89], [90]. As per equation 8, these diversion and mortality values mean that the nets would protect against 85% of all indoor malaria exposure (protection against bites = 100×(1−((1−0.5)×(1−0.7)) %).

ITN coverage in Africa is gradually improving and an increasing number of countries are achieving net coverage of 50% or more, especially for children under fives [29], [37], [91]. To achieve the full potential of nets, including valuable community-wide benefits, it is broadly agreed that reasonably high coverage of entire communities rather than just vulnerable groups is required [36], [43], [92], [93]. Therefore, consistent with the best estimates of the minimum level community-wide coverage required [43], [94], we simulated situations with 50% ITN use across all age groups to represent what is likely attainable in most African countries. In addition, we simulated situations with 80% ITN coverage to represent areas where ITN distribution and coverage in Africa have been highly successful and where existing net distribution and promotion mechanisms may guarantee such coverage levels [90].

Finally, the proportion of normal biting exposure of non-users that occurs indoors when nets would usually be in use (*π_i_*) was set at 0.9 based on recent estimates for *An. gambiae sensu lato* from a malaria-endemic village in south eastern Tanzania [45], [68].

### Parameters describing odor-baited trap technologies

A minimal diversion probability of 0.1 was assumed for mosquitoes encountering odor-baited traps, identical to baseline diversion probabilities from persons not using ITNs and also from cattle. Since there is no possibility of mosquitoes getting blood meals from the odor-baited traps, the probability of successful feeding upon the traps was set to be zero (*φ_t_* = 0). Correspondingly, because traps retain and kill the captured mosquitoes, we set the probability of attack-related mortality upon them to be one (*μ_t_* = 1). Considering the successive stages of host-seeking by a mosquito (Fig. 1), the relative availability of the traps (*λ_t_*) could therefore be varied in different ways.

First, the encounter rate (*ε_t_*) can be increased by making the traps easier for mosquitoes to find, either by placing them in locations close to breeding sites or by improving the attractants (baits) so that the range from which the traps are detected by host-seeking mosquitoes is extended. Moreover, changing the relative attractiveness of the traps to mosquitoes when compared to the attractiveness of actual human hosts, which is equivalent to changing attack probability (*γ_t_*) could also lead to increased or reduced trap catches. However, given the very high attack probabilities assumed in this model, there is little scope for meaningfully increasing this parameter value. It is therefore likely that increasing encounter rates (*ε_t_*) or the number of traps (*N_t_*) are the primary means available to maximize total trap availability (*A_t_*). We therefore hypothesize that these factors represent the key parameters that should be considered when outlining target product profiles for developers of odor-baited traps.

Few studies exist in which odor baits have been compared with humans under realistic field conditions. However in recent field evaluations in rural Tanzania, a mixture of synthetic attractants that mimic human odors, proved to be more attractive than humans to several genera of mosquitoes including malaria vectors [41]. These experimental prototypes attracted approximately four times as many *Anopheles gambiae* as an average human whenever the traps and the human were in separate huts 15 to 100 meters apart, but the humans remained more attractive whenever the two were side by side inside the same hut, resulting in increased exposure of the humans to mosquito bites [41]. This indicates that the synthetic odor blend most probably acts as a long-range cue, attracting more mosquitoes to the point source, at which the mosquitoes then choose the co-located human host based on stronger short-range, non-host-specific stimuli such as heat and water vapor.

These field estimates were therefore used to compute the mean availability of individual traps (*a_t_*) using equation 10 by simply multiplying mean availability of individual humans (*a_h_*) by a factor of four (*λ_t_* = 4). All the relevant intervention parameters and associated values are also outlined in table 4.

### Targeted positioning and delivery systems for odor-baited traps

By comparing the numbers of mosquitoes caught in huts where traps had been placed versus catches in huts where human volunteers slept [41], we estimated the relative availability of the odor-baited traps if such traps are evenly or randomly placed in a set of locations that are geographically distributed in the same way as the human population (*λ_t_* = 4). In such a case of unbiased trap placement among human residences, encounter rates of the traps (*ε_t_*) is simply a function of mean human availability (*a_h_*) and the experimentally measured relative availability of traps (*λ_t_*), which is primarily influenced only by the attractive range of those devices.

For ethical and safety reasons, however, odor-baited traps similar to the ones we have field-tested [40], [41], should never be deployed in such a manner that they are evenly distributed among humans because they emit long-range attractants which can increase exposure of nearby residents for the reasons described above (Sumaye *et al.*, Unpublished). In practice it is impossible to guarantee the minimum distances required to exclude this possibility in even the most modestly clustered human settlements. It is therefore essential that the odor-baited traps are placed far from human residences and aggregations thereof. Fortunately this also offers an excellent means to enhance intervention efficacy and minimize costs.

The targeted placement away from houses is desirable not only to maximize safety but also to take full advantage of mosquito distribution patterns, which naturally present significant opportunities to dramatically enhance effectiveness of mosquito trapping programs. Heterogeneities in the transmission of vector borne infectious diseases including malaria are known to consistently follow the “80/20 statistical distribution” [63] meaning that at least 80% of transmission occurs in 20% or less of all locations. This well established feature clearly implies that deliberately biasing the spatial distribution of any intervention to the most intense foci of vector density, which correspond to locations with higher than average encounter rates and therefore increased availability of the traps, will have correspondingly enhanced impacts upon malaria transmission.

In this model, spatially biasing the location of the traps based on this well-established phenomenon would effectively result in a four-fold enhancement of relative trap availability because with such deliberately biased trap placement, the rates of trap encounter are enhanced four times. Unlike in the case of unbiased placement, the relative trap availabilities (*λ_t_*) are therefore enhanced not only by their longer attractive range, but also by the increased probability that the mosquitoes will encounter those extended odor plumes. It therefore follows that in a situation where these particular traps are biased to locations with 80% of all mosquitoes, their relative availability increased a further four fold, which combined with the field estimates of the enhanced attractiveness yields relative availability of *λ_t_* = 4×4 = 16.

While targeted placement of traps to enhance availability might be achieved by mapping the relevant area and conducting geographic rather than household-based entomological surveys, sufficient resources and institutional capacity to accomplish this are not available in the vast majority of African communities. Nevertheless, we suggest that enough is known about mosquito distribution to enable informal selection of appropriate sites with a reasonable degree of accuracy in most settings that we are familiar with. The kinetic definition of availability, which we have formulated here implies that the availability of the traps for host-seeking mosquitoes will always be higher in areas close to aquatic habitats as this is where the mosquitoes emerge from and also where they return to lay eggs and restart their next host-seeking phase in the beginning of each feeding cycle [61], [65], [95], [96]. Also, houses on the outskirts of aggregated human population such as towns and villages, or around breeding habitats within them [60]–[62], [97], [98] are always exposed to more mosquitoes than those in the centre because mosquitoes dispersing into such settlements inevitably feed predominantly on the hosts they encounter first which are, by definition, more available to them [96].

**Figure 2:**
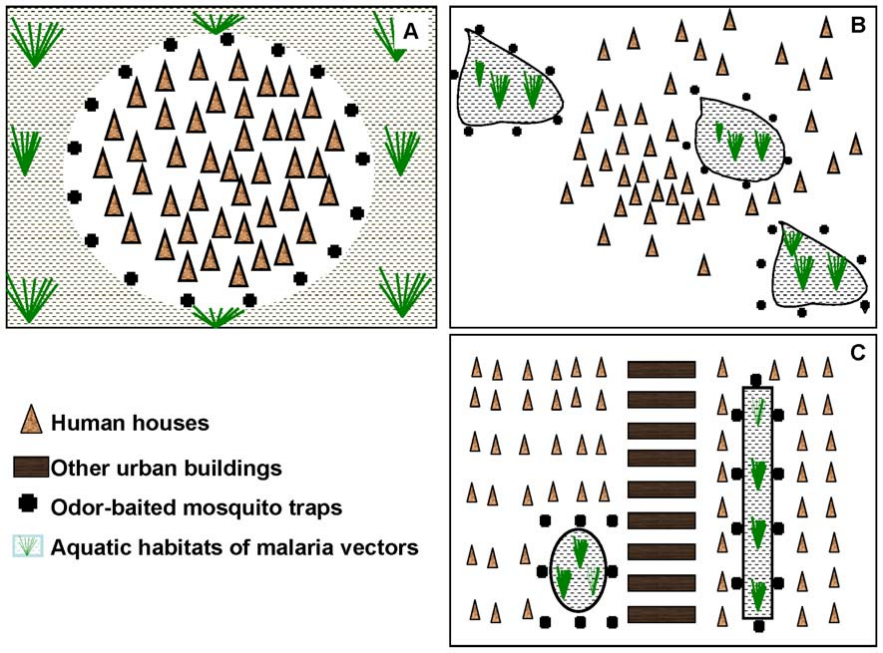
Alternative positional strategies for achieving optimal targeting of odor-baited mosquito traps. The figure shows places where the odor baited mosquito traps should be located in different scenarios namely: **A**; where the communities are small, tightly aggregated and surrounded by large or numerous aquatic habitats, **B**; where habitats are relatively few and easily identifiable such as in arid-rural areas and **C**; in urban settings where the main aquatic habitats are surrounded by human settlements. This diagram is not drawn to scale and is limited to basic structural representations of spatial relationships between human settlements and mosquito larval breeding sites.

This quantitative and qualitative knowledge of mosquito dispersal processes suggests three alternative positioning strategies, which can be implemented even in the absence of fine-scale maps showing mosquito densities, and which can therefore also be used to achieve optimal targeting of the odor-baited traps (Figs. 2A–C). Firstly, where the community is small, tightly aggregated and surrounded by numerous and dispersed aquatic habitats (particularly where these are cryptic or unpredictably distributed) the best solution is probably to surround the perimeter of the settlement with traps (Fig. 2A).

Secondly, where habitats are relatively few in number and easily identifiable, as may be the case in arid rural areas [99], surrounding the breeding sites may offer an even more effective strategy (Fig. 2B). Urban areas where major areas of mosquito proliferation are usually surrounded by human settlement, rather than *vice versa* [97], [98], represent a situation where these two strategies coalesce and are essentially equivalent (Fig. 2C). It should therefore be possible, even without detailed maps of mosquito densities, to selectively position traps in ways that enhance their relative availabilities at least as well as the four-fold increase modeled here.

## Results

**Figure 3:**
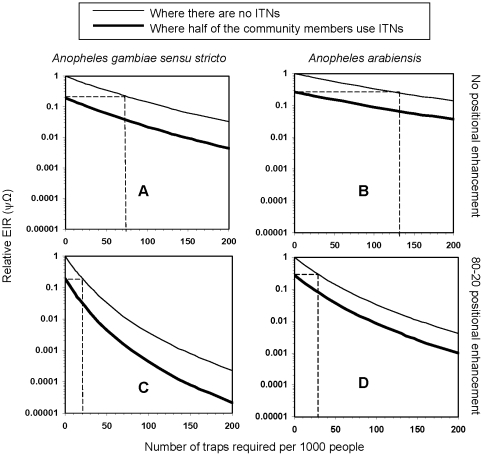
Effects of odor-baited mosquito traps on malaria transmission in situations with moderate ITN coverage. This figure depicts areas where: **A**; the primary vector is *Anopheles gambiae sensu stricto* and the trap locations are not spatially targeted, **B**; the primary vector is *Anopheles arabiensis* and the trap locations are not spatially targeted, **C**; the vector is *Anopheles gambiae sensu stricto* and the trap locations are spatially targeted to satisfy the 80–20 statistical distribution and **D**) where the vector is *Anopheles arabiensis* and trap locations are targeted to satisfy the 80–20 statistical distribution [63]. The dotted lines extrapolate the number of traps per 1000 people that would be required to achieve protection equivalent to ITNs if the traps are used alone. All simulated traps are baited with long-range odors that attract 4 times as many malaria mosquitoes as humans [41].

In all scenarios that we evaluated, odor-baited traps delivered useful levels of protection against malaria exposure with surprisingly few devices required per 1000 people, regardless of whether nets were in use or not (Fig. 3). These simulations indicate that if the traps are baited with long range attractants that are at least four times as attractive to malaria mosquitoes as humans [41], and if they are located in areas where 80% of all mosquitoes are found [63], the traps on their own can confer community-wide protection equivalent to 50% coverage with ITNs.

The number of traps required to achieve these protection levels varies in different scenarios, ranging from 20 units to 130 units per thousand people (Fig. 3). This rate translates to between 1 and 7 traps for every 50 persons, which assuming an average household size of 5, means that at optimum, a single trap would service up to 10 households. Figure 3 also shows that with a similarly modest number of efficient odor-baited traps, malaria transmission can be reduced by 99% or more in these hypothetical scenarios which are representative of most of sub-Saharan Africa. This is expected to occur more readily if the traps are used as complementary intervention alongside ITNs but is nevertheless also plausible if they are deployed as stand-alone vector control methods, especially in places where the primary vector is the anthropophagic *An. gambiae s.s.* (Fig. 3A and C).

**Figure 4 pone-0011573-g004:**
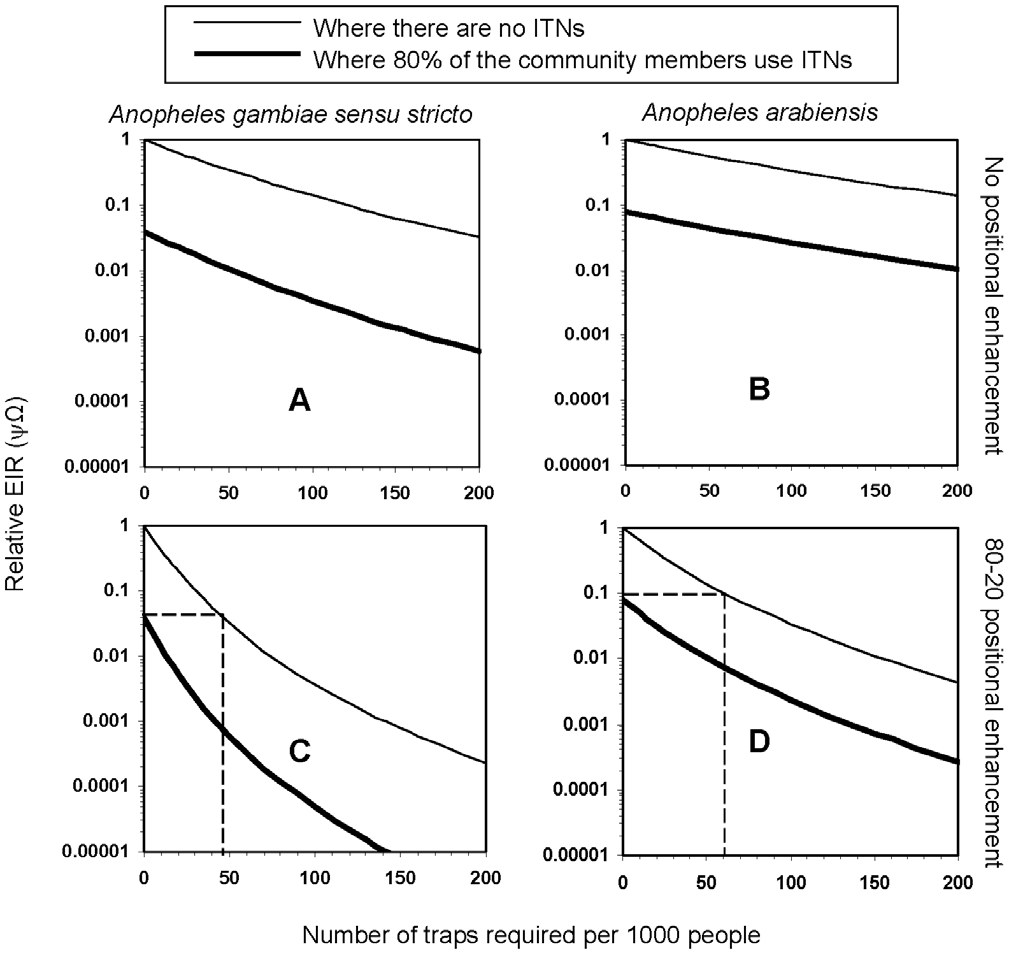
Effects of odor-baited mosquito traps on malaria transmission in situations with high ITN coverage. This figure shows that with high pre-existing ITN coverage (80% in this case), the combined intervention would yield far greater benefits with lower trap numbers than in situations with moderate ITN coverage (for example 50% shown in Fig 3). The dotted lines (not shown in panels A and B) extrapolate the number of traps per 1000 people that would be required to achieve protection equivalent to ITNs if the traps are used alone. All simulated traps are baited with long-range odors that attract 4 times as many malaria mosquitoes as humans [41].

Benefits of such combined interventions are likely to be greater where there is higher pre-existing ITN coverage. It is estimated that, in situations where 80% of community members use ITNs (Fig. 4), malaria transmission could be reduced to far lower limits than in situations with 50% ITN coverage, even though the traps alone may not feasibly match the benefits of such high coverage with ITNs, without geographical targeting. For example, if we consider high transmission situations where unprotected persons are exposed to 200 infections bites per person annually, 80% ITN coverage combined with about 45 traps per 1000 people could reduce relative exposure from 1 to 0.001, meaning an absolute reduction to 0.2 infectious bites per person per year (Fig. 4).

Consistent with previous observations [38] and previous simulations of ITNs [43], [44], malaria transmission by *An. arabiensis* in the presence of cattle can be more difficult to control than transmission in other scenarios because they readily feed upon the cattle, meaning that more vertebrate resources are available to these mosquito populations. Nevertheless, our simulations suggest that integrated vector management packages consisting of ITNs and odor-baited traps will still drastically reduce transmission in these situations. Figs. 3B and 3D show that, so long as the availability of traps is enhanced by spatially targeted positioning, as few as 30 traps per 1000 people can achieve protection equivalent to 50% ITN coverage, even where such alternative hosts are available to the malaria vectors.

**Figure 5:**
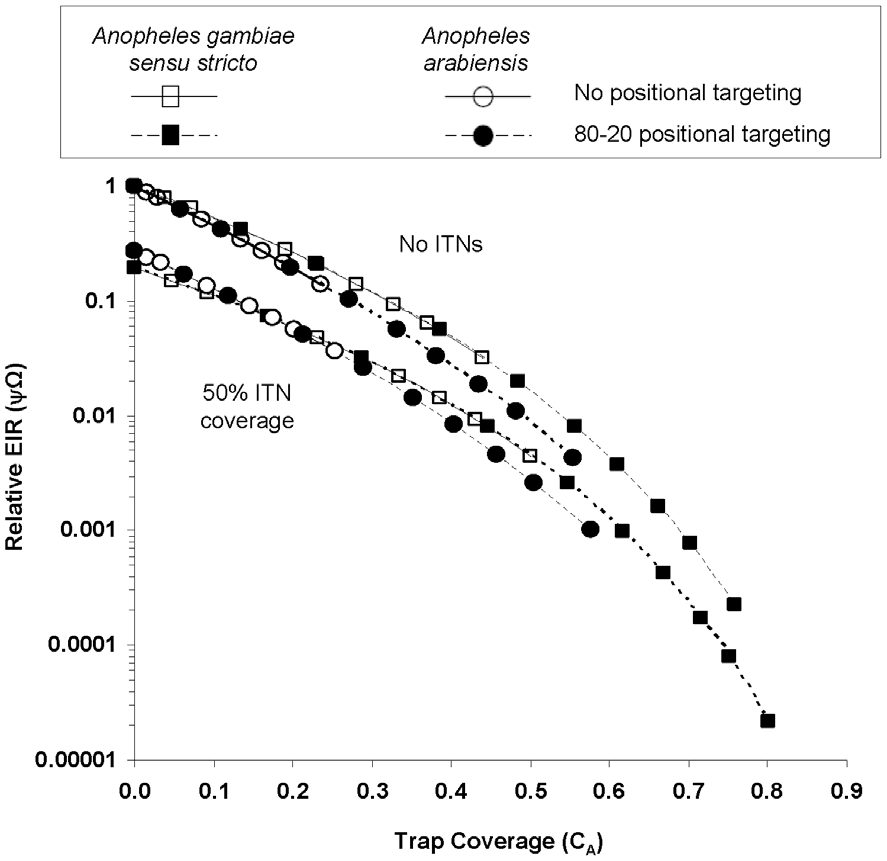
Relationship between trap coverage (C_A_) and relative malaria exposure (Ψ_Ω_). This figure shows predicted relationship between proportion of total availability of hosts and pseudo hosts that is accounted for by odor-baited traps (trap coverage; C_A_) and resulting relative exposure to malaria (Ψ_Ω_) when odor-baited mosquito traps are used in communities where there are no ITNs or in communities where half of the population already uses ITNs. The simulated traps are baited with long-range odors, which can attract at least four times as many malaria mosquitoes as humans [41]. The trap coverage (C_A_) can be improved by several means, for example by increasing bait attractiveness, biasing trap locations towards areas with most mosquitoes, increasing the number of traps, or removing cattle from the area. Spatial targeting according to the 80–20 statistical distribution means concentrating the traps in areas where at least 80% of all mosquitoes are found [63]. All data points presented here are sampled from the simulations described in figures 3 and 4.

Benefits of odor-baited traps as a tool against malaria arise from their function as decoy hosts, which do not provide any blood but capture host-seeking mosquitoes that attack them. Figure 5 shows that malaria transmission is expected to decline drastically and exponentially in response to increases of the proportional contribution of the traps, to the total availability of all hosts and pseudo-hosts that can be attacked by host-seeking malaria mosquitoes (*C_A_*).

This term *C_A_*, is best thought of as the coverage of all available host types with the trapping devices or the proportion of total host availability (*A*) that they account for. As the trap coverage (*C_A_*) increases, EIR decreases dramatically and exponentially, regardless of the vector-host combinations or whether ITNs are used or not (Fig. 5). The consistency of this trend across scenarios suggests that increasing individual trap availability by enhancing either the long-range attractiveness of these devices, increasing the number of traps, or by targeting the traps to the foci of highest mosquito density, is crucial to maximizing the epidemiological impact and/or minimizing the cost of this technology. It also elucidates a clear quantitative rationale for the attenuated impact of ITNs and traps upon vectors like *An. arabiensis*, which have alternative non-human hosts: such mosquito populations can exploit blood resources from a larger quantity of available hosts so a correspondingly greater quantity of traps are required to compete with the available natural hosts.

Lastly, as may be logically expected in nature, the simulations show that various mosquito feeding cycle processes and events that determine malaria transmission by the vector are reduced when odor baited traps are introduced, and when the number of traps is increased. For example, the feeding cycle length, the host seeking interval, and also the probability of surviving one complete feeding cycle, are all reduced (File S1).

## Discussion

Using an adapted and conceptually reformulated mathematical model, we have successfully determined that odor-baited mosquito traps could potentially provide substantial protection against malaria risk in various epidemiological scenarios in sub-Saharan Africa. We have shown that even if existing coverage with insecticidal nets were 50%, traps could dramatically augment the benefits of ITNs. Although the simulated odor-baited mosquito traps can deliver encouraging levels of protection even when used on their own, the benefits are far greater when the traps are deployed to complement rather than to replace the ITNs (Figs. 3–5). This theoretical evidence reinforces the view that odor-baited traps could have genuine potential for malaria vector control [100], [101] in Africa, where most of the present day malaria burden exists [78], [91].

While this work encouragingly predicts that odor-baited traps might be developed into valuable tools for malaria transmission control, the simulated example is based on field evaluations of an experimental prototype [41], which would be prohibitively expensive for community-level scale-up or even large-scale efficacy trials. Improved, cost-effective trap models which translate such theoretical optimism into practical realization of malaria control therefore remain a future ambition to be pursued. While some progress has recently been made towards this goal [40], [42], much remains to be done.

Perhaps the most useful outcome of this modeling exercise is therefore the identification of key characteristics that will determine the cost-effectiveness of these technologies, including how best they should be positioned and how best they may be delivered as a health commodity. First of all, the traps should be fitted with super-attractive odor lures, which can attract more mosquitoes than normal vertebrate hosts. Even though our simulations considered traps baited with long-range lures that attract 4 times as many mosquitoes as humans, high trap coverage (*C_A_*) values can be obtained even with baits that have lower degrees of attractiveness, so long as targeting of the traps to appropriate locations is proportionately enhanced by placing them in areas where mosquitoes are most abundant, or by simply using more traps. Developers of odor-baited trap technologies should therefore focus on odor baits that attract at least as many mosquitoes as real humans.

The other important characteristic is financial cost of the technology. If odor-baited traps were to be promoted for malaria control in Africa, they would need to at least match the cost-effectiveness of ITNs, which apart from being one of the primary interventions, are also one of the most cost-effective health commodities in existence, comparable with childhood vaccinations [102], [103]. The most recent estimates based on 5 large-scale distribution programmes for insecticidal nets indicate it costs approximately 2005 US$2.10 (Range 1.46 to 2.64) to provide one year of protection with a treated net [104].

Even assuming that each ITN is used by only one person so that 500 would be required to achieve 50% coverage of our simulated population of 1000, the 20 to 130 traps required to provide equivalent protection (Fig. 3) would have to cost a maximum of 2005 US$52.45 to $8.07 per trap per year, respectively, to achieve equivalent cost effectiveness (File S1). If we now consider that ITNs are commonly used by more than one person and adjust accordingly (mean of 1.9 occupants per net in the field setting where these trap prototypes were evaluated [105]), the standards of cost-effectiveness set by ITNs are even more challenging to match: Even if only 20 traps per 1000 people is sufficient, each would have to cost a maximum of 2005 US$27.61 per annum for total costs of procurement, transport, installation, operation, maintenance while the less tractable *An. arabiensis* dominated scenario requiring 130 traps per 1000 people indicates a maximum cost of $4.25 per annum (File S1).

Such low deployment costs are a lot to ask of any technology or implementation program and should be carefully considered by developers of odor-baited technologies for malaria transmission control. Developing a sufficiently cost-effective trap is probably the greatest technical hurdle this strategy must overcome to become a realistic option for malaria control programmes across Africa. Even if all the other necessary characteristics were fulfilled, developing devices which can affordably produce sufficient quantities of CO_2_, the only bulk attractant in the current prototypes [41], is most probably the greatest challenge ahead. The experimental prototype of the odor-baited traps that we have considered here, as well as simpler more recent designs [40], [42], remain far too expensive to consider at this stage for future large-scale use. In addition to the need for cheaper CO_2_ generation, it also follows traps should be small and practical enough to be delivered and maintained in isolated African villages at reasonable costs.

Unlike ITNs which can be marketed as household consumer products, traps provide only communal benefits and would require a customized delivery mechanism to maximize its usefulness. We expect that even if the target product profiles that we have outlined here were manageable cost-wise, vertical and presumably community-based delivery mechanisms would be necessary to supply and deploy the traps. We propose that where local governance and administrative systems are already strengthened, or where they can be supported by centralized national malaria control programmes, sustainable implementation of a traps-based strategy may possibly be achieved through participatory approaches similar to those applied for scaling up community-based sanitation technologies like Ventilated Improved Pit (VIP) latrines or water source protection among rural communities in developing countries [106]–[109].

We are not aware of any large scale malaria vector control operations which have used traps of any nature and with which we could directly compare our simulation results. Perhaps the most similar example is the 1980s tsetse fly control program in Zambezi valley, in Zimbabwe, where up to 3000 odor-baited tsetse fly targets treated with insecticides were deployed in an area of 600 square kilometres [14]. Considering the trap requirements predicted by our model, and comparing the simulated scenarios to this particular Zambezi valley tsetse fly program [14], it can be argued that traps might indeed be a viable option for further industrial development to combat malaria.

An obvious aspect of the outlined target product profile is that some of the essential trap characteristics can be traded off against each other. This is encouraging because such trade-offs may be undertaken to minimize costs of manufacture, installation or maintenance of the traps. For example, instead of super-attractive lures that may be too expensive to obtain, one may opt for moderately attractive lures but use larger numbers of more affordable traps and/or ensure that the trap positioning is enhanced.

None of these simulations would have been possible without reconsidering the fundamental biological definition of what an available host is and distinguishing this from the availability of blood. While host availability has been defined as either of these two possibilities (attackable hosts [52] versus blood [43], [44] in previous models), this is the first time that this crucial distinction has been explicitly considered and separately parameterized. The combination of ITNs with odor-baited traps proved an ideal example because, while the former has a non-zero value for both parameters, traps provide no blood and cannot be plausibly represented with models which do not distinguish between these two quantities. Beyond this specific application, this fundamental re-evaluation of how resource acquisition processes can be conceptualized may be particularly useful for modeling intervention options as diverse as mosquito repellents [110], [111], house screening [112] and the auto-dissemination of larvcides [113] and slow acting adulticides [114].

Recent advances in mathematical modelling of how agricultural pests interact with pheromones suggest that such kinetic approaches could greatly improve evaluation of various interventions that use synthetic odor-cues, including not only host-derived attractants, but also pheromones usually used to disrupt insect mating in agricultural fields. For example in a recent publication by Miller *et al*, in which simple algebraic equations for attraction and competitive attraction were validated, *cumulative moth catches* were expressed as a function of *findability of trap baited with pheromone lures*, *efficiency of the traps*, the *retention time of the moths in the traps* and the *densities in an environment* [115]. If compared to the host-seeking processes of female mosquitoes as presented in this paper, *findability of traps* as presenter by Miller et al [115] may be considered analogous to *trap encounter rates* (Eq. 1 of this paper), while, *trap efficiencies* would be set to 1.0, with an infinite retention time of all mosquitoes that attack the traps, assuming that trapped mosquitoes do not escape afterwards. Nevertheless, it may be stated also that the current analyses deals more with competitive attraction, as opposed to non-competitive attraction, and that odor-baited mosquito traps must therefore have relative availabilities greater than 1.0, so as to be effective.

Though we consider these simulations to have been generally successful, we also recognize that there were some limitations with this particular model. For example, it is assumed that at the point when the vector attacks the host, there are only two possibilities: that either the vector feeds successfully and consequently survives or it dies in the attempt before obtaining a blood meal (Eq. 4). This argument implies that no mortality occurs after blood meal acquisition, and instead considers all attack related mortality as occurring prior to feeding. This is not entirely true since there can be additional mortality immediately after feeding or midway through feeding, by which time malaria transmission may have occurred if the host was a susceptible human. As such, the model may slightly underestimate effects of ITNs on mosquito mortality. We therefore advise that our results be interpreted in view of protection from human exposure to infection as the model may not capture the full impact of ITNs on onward transmission, mediated by mosquitoes picking up parasites from a protected person and successfully transmitting the parasites to another person. Also, as has been the case with essentially all the deterministic malaria transmission models, with a few notable exceptions [51], [96], [116], our formulation does not consider fine scale spatial relations and heterogeneities in the dynamics of mosquito and human populations.

Lastly, it should be noted that in order for our findings to be generalizable to different transmission scenarios across Africa, this model formulation and also its previous versions [43], [44] use relative EIR on a log scale of 0 to 1 instead of empirical field estimates, to represent various outcomes of the modelled interventions. We recognize however, that for each individual scenario, it would be more reasonable to use absolute empirical indicators, such as mosquito trap catches, or malaria parasite prevalence rates. As such our simulations and findings do not exclude the essential need for field evaluation, by way of community scale trials, to ascertain the actual benefits of combining ITNs with odor-baited mosquito traps.

Nevertheless, these simulations do allow for much clearer quantitative insights into the future potential of odor-baited mosquito traps strategies for malaria transmission control.

### Conclusions

Odor-baited mosquito traps could provide substantial protection against malaria in their own right and could augment benefits already achieved with ITNs if deployed as a complementary intervention. For this strategy to succeed, we propose that the following three key criteria should be met: 1) that the odor-baits should be considerably more attractive to malaria vectors than humans, 2) that the traps should be located in areas where host-seeking mosquitoes are concentrated and 3) that they need to be cheap and easy to deploy at a rate of 20–130 traps per 1000 people. Finally, if efficacious interventions matching this target product profile were developed, we recommend that the most appropriate way to deploy them effectively and sustainably would be through vertical rather than horizontal delivery mechanisms, which will require strong technical support from central authorities such as National Malaria Control Programmes, as well as broad progress towards improved governance and capacity of local authorities to implement such programmes on the ground.

## Supporting Information

File S1 Model worksheet showing various simulated scenarios. This file contains the model in a Microsoft Excel spreadsheet where all the simulations can be regenerated. Included in the workbook is an additional sheet containing comparative costings of odor-baited mosquito traps versus insecticide treated nets.
(1.41 MB XLS)
